# Bridge Structure Deformation Prediction Based on GNSS Data Using Kalman-ARIMA-GARCH Model

**DOI:** 10.3390/s18010298

**Published:** 2018-01-19

**Authors:** Jingzhou Xin, Jianting Zhou, Simon X. Yang, Xiaoqing Li, Yu Wang

**Affiliations:** 1 School of Civil Engineering, Chongqing Jiaotong University, Chongqing 400074, China; xinjz@mails.cqjtu.edu.cn (J.X.); 622160086010@mails.cqjtu.edu.cn (X.L.); 2 School of Engineering, University of Guelph, Guelph, ON N1G 2W1, Canada; 3 School of Engineering, Cardiff University, Cardiff CF24 3AA, UK; WangY219@cardiff.ac.uk

**Keywords:** bridge engineering, deformation prediction, structural health monitoring, bridge sensor data

## Abstract

Bridges are an essential part of the ground transportation system. Health monitoring is fundamentally important for the safety and service life of bridges. A large amount of structural information is obtained from various sensors using sensing technology, and the data processing has become a challenging issue. To improve the prediction accuracy of bridge structure deformation based on data mining and to accurately evaluate the time-varying characteristics of bridge structure performance evolution, this paper proposes a new method for bridge structure deformation prediction, which integrates the Kalman filter, autoregressive integrated moving average model (ARIMA), and generalized autoregressive conditional heteroskedasticity (GARCH). Firstly, the raw deformation data is directly pre-processed using the Kalman filter to reduce the noise. After that, the linear recursive ARIMA model is established to analyze and predict the structure deformation. Finally, the nonlinear recursive GARCH model is introduced to further improve the accuracy of the prediction. Simulation results based on measured sensor data from the Global Navigation Satellite System (GNSS) deformation monitoring system demonstrated that: (1) the Kalman filter is capable of denoising the bridge deformation monitoring data; (2) the prediction accuracy of the proposed Kalman-ARIMA-GARCH model is satisfactory, where the mean absolute error increases only from 3.402 mm to 5.847 mm with the increment of the prediction step; and (3) in comparision to the Kalman-ARIMA model, the Kalman-ARIMA-GARCH model results in superior prediction accuracy as it includes partial nonlinear characteristics (heteroscedasticity); the mean absolute error of five-step prediction using the proposed model is improved by 10.12%. This paper provides a new way for structural behavior prediction based on data processing, which can lay a foundation for the early warning of bridge health monitoring system based on sensor data using sensing technology.

## 1. Introduction

As the key node of interoperability of traffic systems, the bridge is a vital guarantee for the development of the economy and social safety. Bridge construction plays a crucial role in resolving overcapacity of traditional industries, promoting the development of new strategic industries and the third industry, boosting the integration and upgrading of industries, and stimulating economic growth [1]. With the continuous development of sensing technology and information science, health monitoring systems are widely used in the safety guarantee of the bridge structure. As a paramount monitoring index that reflects the overall stiffness of the structure, deformation is the macro response of the bridge micro complex mechanical mechanism, which contains the internal mechanical evolution information of the structure and affect the safety of bridge structure significantly. Thus, predicting the structural deformation is of great scientific significance and engineering application value in order to give full play to the early warning benefit of the health monitoring system, as well as the structural safety of the bridges [2].

For the prediction of structural deformation, many studies have been conducted, which can be broadly classified into two categories. One is the prediction method based on the mechanical mechanism, which emphasizes the deterministic function relationship between cause and effect, and is a predictive model with a priori characteristic. For instance, Liu et al. [3] established a shrinkage and creep model of high crack resistance and compact concrete. The method for forecasting a long time structural behavior of Hong Kong-Zhuhai-Macao Bridge was obtained by adding and accumulating the increment of strain in every minute period for 30 years. Sajedi et al. [4] proposed an analytical procedure that can predict the flexural behavior of intact and corroded reinforced concrete beams, with or without lap splices considering the bond stress-slip behavior at the steel–concrete interface. Han et al. [5] built a new computational framework for Bayesian inference regarding the long-term deflection of concrete structures, importance sampling technology, and response surface approach, and a stochastic process was introduced to improve computational efficiency and describe the random properties of creep. As the solution of nonlinear differential equations of complex structures is arduous to realize, the above-mentioned prediction methods often rely on numerical simulation models. However, the prediction results of this model need to be further verified due to the complex and changeable bridge service environment, the immature constitutive theory of concrete, and the high randomness and uncertainty of material parameters that appear with an increase of service life. Besides, the performance evolution mechanism of different structural forms is different, which leads to the universality of this method. The second type is the method based on data mining of monitoring information. The method can make full use of the macro response information of the actual structure, and avoids the complex internal mechanism of structural deformation. It is an effective approach to dynamic control of structural information; such as linear models, for example, the autoregressive moving average model (ARMA) [6,7] and autoregressive integral moving average model (ARIMA) [8,9]; and nonlinear models, such as the artificial neural network method (ANN) [10,11,12], extreme learning machine (ELM) [13,14], support vector machines (SVM) [15], and ant colony optimization algorithm (ACO) [16]. The research shows that the monitoring data of large structures has nonlinear and non-stationary characteristics because of the ambiguous service environment [10]. The linear models are only capable of stationary linear or simple non-stationary linear time series prediction, which are hard to express in terms of deformation time series with high nonlinear and non-stationary characteristics, and linear versions can only get limited prediction accuracy [17]. ANN has some limitations, such as easy oscillations and slow convergence speed [18]. It is arduous to determine the key parameters and avoid the subjectivity caused by artificial selection of ELM, SVM and ACO [19,20,21]. 

In recent years, the structural time series model (STM), fusing state space model and Kalman filter, gets a growing concern because of its excellent prediction capability. The structural time series model was first proposed by Harvey, a British econometrics economist, in 1983 [22], and the principle of STM was formally presented and elaborated in his monograph in 1989 [23]. The structural time series model decomposes the original time series into a variety of random components, such as trend, cycle, seasonal, irregular and so on. The structural time series model has a natural form of state space. It does not only express the unobservable components by using state vectors, but also estimates, smoothes and predicts every component of the state vector based on the Kalman filter, which is widely applied in economic and non-economic fields [24,25,26]. Aamir et al. proposed an ARIMA-Kalman model for forecasting Pakistan’s monthly crude oil price [27]; Sebasti’an et al. presents for the first time a state space representation for generalized autoregressive conditional heteroscedasticity model (GARCH) family of time series models and proposes a novel estimation procedure based on the extended Kalman filter [28]. In the realization aspect, Ian et al. provide a summary of a selection of the high-quality published computational time series research using R [29]. The above research promotes the theoretical development and practical application of the STM model. In the field of bridge health monitoring, Omenzetter et al. formulate a vector seasonal autoregressive integrated moving average model for the recorded strain signals [30]. The coefficients of the ARIMA model are allowed to vary with time and are identified using an adaptive Kalman filter, and the time series model here is mainly used for structural damage identification as well as other research [31,32,33]. The method of combining time series with Kalman filter is not yet applied for deformation prediction.

Due to the difficulty of structure deformation prediction as well as the insufficiency of the existing method, a new federated deformation prediction method is established by integrating Kalman filter, ARIMA and GARCH model based on the time series essence of health monitoring data. Unlike STM, the time series model in this paper is a traditional Box-Jenkins time series model, and the main function of Kalman filter here is to reduce the noise of the initial data. The performance of this proposed method is demonstrated on a long span urban rail-transit cable-stayed bridge. Compared with the prediction accuracy of the ARIMA model, the proposed method can improve the accuracy of the monitoring data of a complex bridge and achieve superior prediction results.

## 2. Multiple Sensors Used for Bridge Structure Deformation Prediction

The paper is organized as follows. In Section 2.1., a health monitor system, including various sensors of a real bridge, is stated. In Section 2.2., a Global Navigation Satellite System (GNSS) kinematic deformation monitoring system is further introduced, and in Section 2.3., the principle of GNSS is provided.

### 2.1. Bridge Sensors

**Figure 1:**
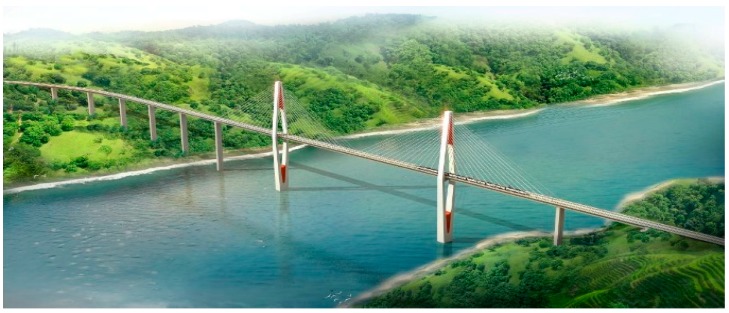
The Caijia Jialing River Bridge.

**Figure 2:**
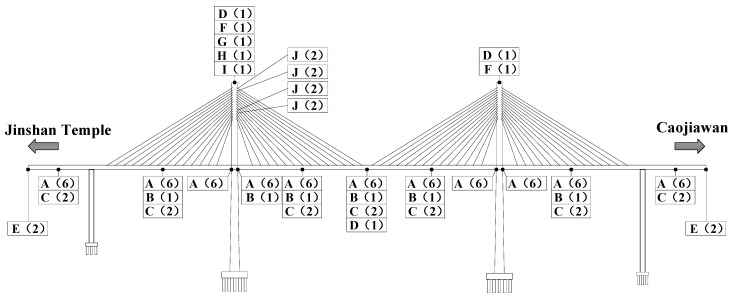
The overall layout of the sensor measuring point: **A**, the stress sensor (including temperature measurement as well); **B**, the acceleration sensor; **C**, the static level gauge; **D**, Global Navigation Satellite System (GNSS); **E**, Linear Variable Displacement Transducer; **F**, the tiltmeter; **G**, the temperature and humidity sensor; **H**, the pluviometer; **I**, the dogvane and anemoscope and **J**, the anchor cable meter through the cable. Also, the numbers in parentheses indicate the amount of each sensor.

The Caijia Jialing River Bridge, located in Chongqing, China, is the second longest rail transit cable-stayed bridge in the world. The total length of the bridge is 1250 m, and the main structure is a dual-pylon dual-cable plane concrete cable-stayed bridge with rhomboic towers (Figure 1). The layout of spans is 60 m + 135 m + 250 m + 135 m + 60 m = 640 m. The bridge had established a comparatively comprehensive bridge health monitoring system to ensure the safety of serviceability, durability, and sustainability of the bridge. The overall layout of sensors is shown in Figure 2.

Combined with the construction progress of the bridge, the health monitoring sensors were appropriately installed at the corresponding positions as follows.

**Figure 3:**
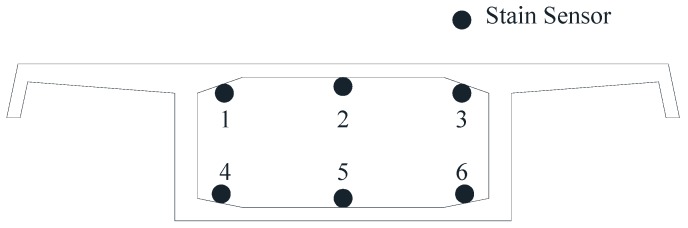
The layout diagram of strain sensors on the surface of concrete.

(1) Main girder. Along the longitudinal direction of the bridge, the vibrating wire strain sensors were attached to the lower edge of the top slab and the upper edge of the bottom slab, which are shown in Figure 3. It is worth mentioning that the sensor could also measure the temperature of the structure and modify the initial temperature. Also, static level gauges based on the pipe principle were installed to monitor the static long-term deformation of the girder. Meanwhile, the GNSS was installed to monitor the spatial mid-span deformation. Also, the displacement sensors and the acceleration sensors were installed to measure the width of the expansion joint and to test the dynamic characteristics, respectively.

(2) Stay-cable. Intelligent anchor cable meters with temperature measurement included were embedded to monitor the cable force before the cable tension in the construction stage and anchorage of the cable and tower. The intelligent six-string meters were applied for measuring the cable force under the eccentric load to ensure the accuracy.

(3) Main tower. The tiltmeters and GNSS were installed on the top of the tower, and a monitoring station was established for monitoring meteorological factors including wind speed, wind direction and rainfall, and humidity.

### 2.2. Global Navigation Satellite System Kinematic Deformation Monitoring System

**Figure 4:**
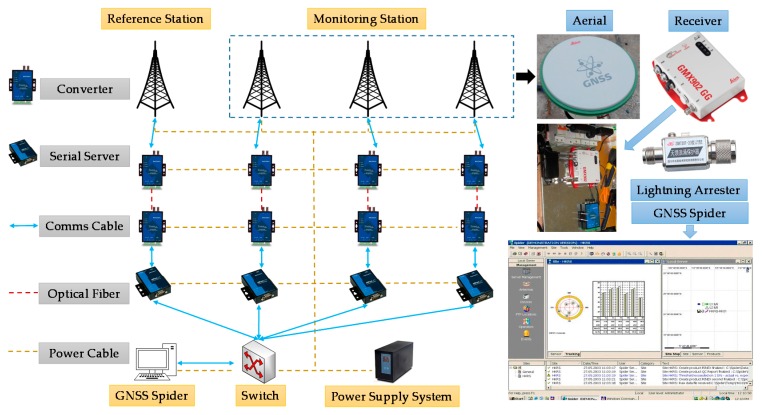
GNSS deformation monitoring system.

To measure the deformation of the pivotal components of the bridge, the GNSS kinematic deformation monitoring system was applied, which consists of a data acquisition system, data transmission system, lightning protection system, control and analysis system, and power system. A total of four stations were set up, including one reference station established in the non-deformation area, and three monitoring stations located at the top of the two main towers and the mid-span, respectively. Considering the impact of train operation on GPS, the mid-span measurement point was placed on the outside of the lateral deck. Dual frequency double star monitoring receiver GMX902GG (Leica Geosystems AG, St. Gallen, Switzerland) and antenna AS10 (Leica Geosystems AG) are applied in the four measuring stations. The data from the stations are transmitted by fiber optics transmission independently. The control and analysis system realizes the automation of data observation, data decoding and processing, and equipment management by GNSS Spider (Leica Geosystems AG). The working network of GNSS deformation monitoring system is shown in Figure 4.

### 2.3. Principle of Global Navigation Satellite System Kinematic Deformation Monitoring

Global Navigation Satellite System refers to all satellite navigation systems, including American GPS, Russian Glonass, European Galileo, and China’s Beidou navigation systems and related enhancement systems. There are many shared characteristics in the independent satellite system, for instance, each system consists of a ground control component, space satellites and a user component. The satellite signal contains three levels, namely, carrier, pseudo code and navigation message data code [34]. The main functions of ground control component are tracking GNSS satellites, determining the orbits of satellites and satellite clock parameters, forecasting and compiling navigational messages. The main function of the space satellite is to continually broadcast the ranging signals and navigation messages for navigation and positioning so that the earth can receive a sufficient number of satellite signals at any time. The navigation message includes satellite clock and satellite orbit, etc. 

One of the key problems of GNSS positioning is how to determine the distance from the satellite to the ground sensor. The methods including the code pseudo measurement and the carrier phase measurement are typically adopted, and carrier phase measurement is the one to achieve higher precision positioning. The GNSS signal carrier is a cosine wave with a short wavelength. The wavelength of two frequencies is 19.0 cm or 24.4 cm [34]. The measurement error at the level of mm is completely acceptable in the monitoring of large bridge structure [35]. The observation equation of carrier phase measurement can be constructed by

(1) $$\varphi_{i}\lambda =\sqrt{{\left(X^{i}-X\right)}^{2}+{\left(Y^{i}-Y\right)}^{2}+{\left(Z^{i}-Z\right)}^{2}}-cV_{i_{R}}+cV_{i_{i}s}-N_{i}\lambda -{\left(V_{\mathrm{ion}}\right)}_{i}-{\left(V_{\mathrm{trop}}\right)}_{i},$$

where i=1,2,3…, and i represents the satellite number; λ is the carrier wavelength; $\varphi_{i}$ represents phase difference which less than an entire cycle; $\left(X^{i},Y^{i},Z^{i}\right)$ is the location of the satellite in space based on the satellite ephemeris; (X,Y,Z) is the location of the GNSS sensor; c is the speed of light; $V_{i_{R}}$ and $V_{i_{i}s}$ are the receiver clock error and satellite clock error, respectively; $V_{\mathrm{ion}}$ and $V_{\mathrm{trop}}$ are ionospheric delay and tropospheric, respectively; and $N_{i}$ is the ambiguity of whole cycles.

## 3. Prediction Model

In this section, a brief review of the Kalman filter, time series model, and GARCH will be presented, respectively.

### 3.1. Kalman Filter

Due to the complexity of the bridge structure monitoring environment, there may be a significant amount of random noise in the observation data—the deformation curve shows the characteristic of a small value with a large fluctuation. In this study, the optimal estimation Kalman filter algorithm in the least mean square sense is used to de-noise the random noise of the data. In most cases, the discrete Kalman filter is the major application types [36]. The mathematical model is as follows:

(2) $$x_{k}=F_{k|k-1}x_{k-1}+w_{k},x_{k},x_{k-1}\in R^{\eta },$$

(3) $$y_{k}=H_{k}x_{k}+v_{k},y_{k}\in R^{\varpi },$$

where $R^{\eta }$ and $R^{\varpi }$ represent η- and ϖ-dimensional real variable domains, respectively; $x_{k}$ and $x_{k-1}$ are the state vectors at steps k and k−1, respectively; $y_{k}$ is the observed measurement value at step k; $F_{k|k-1}$ is the transition matrix; and $H_{k}$ is the measurement matrix. Both transition and measurement matrices can be variable matrices or constant matrices. Vectors $w_{k}$ and $v_{k}$ represent the process and measurement noises, respectively. They are assumed to be additive, white, and independent of each other, and have the probability distribution p(w)~N(0,Q) and p(v)~N(0,R). Let ${\hat{x}}_{k}^{-}$ and ${\hat{x}}_{k}$ represent the a priori and a posteriori state estimates at step k, respectively, where the error covariance matrices are calculated by

(4) $$P_{k}^{-}=E[(x_{k}-{\hat{x}}_{k}^{-}){\left(x_{k}-{\hat{x}}_{k}^{-}\right)}^{T}],$$

(5) $$P_{k}=E[(x_{k}-{\hat{x}}_{k}){\left(x_{k}-{\hat{x}}_{k}\right)}^{T}].$$

**Figure 5:**
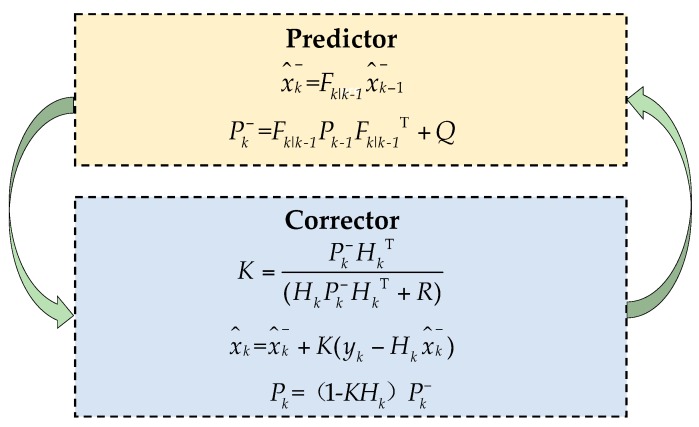
Predictor-corrector algorithm of Kalman filter.

The Kalman filter uses a predictor-corrector algorithm to estimate $x_{k}$ as shown in Figure 5 [36]. Firstly, a tentative estimate ${\hat{x}}_{k}^{-}$ is calculated based on the value of ${\hat{x}}_{k-1}^{-}$, then the measurement value $y_{k}$ is used to further refine the value of ${\hat{x}}_{k}^{-}$ in order to obtain ${\hat{x}}_{k}$, which is the estimate of $x_{k}$

### 3.2. Test for Stationary of Time Series

In this paper, the runs test using to check the stationarity of time series is conducted, then the corresponding model is established according to the test results. The specific steps of the run test are shown below:

Step 1: Calculate the mean value $\bar{X}$ of sample series X(t).

Step 2: Code values above $\bar{X}$ as positive and values below $\bar{X}$ as negative. Thus, a sequence of symbols corresponding to the original series can be obtained. Each consecutive sequence of the symbolic sequence is defined as a run, and the total number of runs is r.

Step 3: For random sequences, assume the length of the sequence is N, and $N_{1}$, $N_{2}$ reflect the number of positive and negative occurrences in the sequence, respectively.

Step 4: The stationarity of a random sequence X(t) can be tested by the following equations:

(6) $$Z=\frac{r-E(r)}{\sigma (r)},$$

(7) $$E(r)=2N_{1}N_{2}/N+1,$$

(8) $$\sigma (r)={\left[\frac{2N_{1}N_{2}(2N_{1}N_{2}-N)}{N^{2}(N-1)}\right]}^{1/2}.$$

As the statistic *Z* obeys the normal distribution approximately, at the 5% significance level, a test statistic with an absolute value greater than 1.96 indicates non-stationary.

### 3.3. Time Series Model

A time series model can be classified into two categories: a stationary model, such as autoregressive model (AR), moving average model (MA), ARMA; or a non-stationary model, such as ARIMA. 

For non-stationary deformation time series, ARIMA(p,d,q) model should be adopted; ARIMA(p,d,q) can be constructed below,

(9) $$\phi (B){\left(1-B\right)}^{d}X(t)=\theta (B)a(t),$$

(10) B=X(t−1)/X(t),

(11) $$\phi (B)=1-\phi_{1}B-\phi_{2}B^{2}\cdots -\phi_{p-1}B^{p-1}-\phi_{p}B^{p},$$

(12) $$\theta (B)=1-\theta_{1}B-\theta_{2}B^{2}\cdots -\theta_{q-1}B^{q-1}-\theta_{q}B^{q},$$

where X(t) represent the measured deformation for the moment t (t=1,2,3,⋯), while a(t) is the residual errors at the same moment, which should satisfy the Gaussian white noise process with a mean value of 0; $\theta_{j}(j=1,2,3,\cdots q)$ is the parameters to be estimated for the model; B is the difference operator; and p, d and q represent autoregressive order, difference order, and moving average order of the model, respectively. In particular, when *d* = 0, the ARIMA(p,d,q) model is equal to the ARIMA(p,q) model.

### 3.4. Explanation and Test Method of Heteroscedasticity

One of the critical hypotheses of the classical linear regression model is that the random disturbances have equal variance, which guarantees unbiased, effective and consistent regression coefficients. In other words, they assume that the data is homoscedastic. When the hypothesis is not well-established, the validity and consistency of the regression estimation coefficient cannot be guaranteed, which leads to the estimation bias of the regression coefficients and lower fitting precision of the model. To cope with the above problems, Engle proposed the autoregressive conditional heteroskedasticity model (ARCH) [37]. The basic idea is that the residuals obey the Gaussian distribution whose mean is zero and variance is a time-varying variance (conditional heteroscedasticity), and the variance can by expressed by a linear combination of the squared residuals of the past limited term. The essence of the model is fitting the current heteroscedastic function values by using the finite moving average term of the squared residual sequence. However, in many cases, many heteroscedastic functions of residuals have a long-term correlation, if fitting heteroscedasticity functions using the ARCH model will produce high moving average order, the difficulty of parameters estimation increased. For that reason, Bollerslev proposed GARCH [38], which defines the current heteroscedasticity function as a weighted combination of past heteroscedasticity functions and the squared residuals of the past. The number of parameters in the model and the difficulty of parameter estimation are reduced. Meanwhile, the processing ability of heteroscedasticity is further improved. At present, the intuitionistic methods of testing heteroscedasticity include the residual plot test, residual square test, and analytic method.

### 3.5. Generalized Autoregressive Conditional Heteroskedasticity Model

The mathematical form of the GARCH model can be expressed by

(13) $$X(t)=\phi_{1}X(t-1)+\cdots +\phi_{p}X(t-p)+a(t)+\theta_{1}a(t-1)+\cdots +\theta_{q}a(t-q),$$

(14) $$a(t)=\sqrt{h_{t}}e_{t},$$

(15) $$h_{t}=\beta_{0}+\sum_{u=1}^{m}\beta_{u}a_{t-u}^{2}+\sum_{v=1}^{n}\alpha_{v}h_{t-v},$$

where the mean Equation (13) can be obtained by the ARIMA model; $\left\{a_{t}\right\}$ is the residual sequence; and $\left\{e_{t}\right\}$ represents an independent and identically distributed random sequence with mean 0 and variance 1. It usually has three kinds of distributions, that is, standard normal distribution, *t* distribution and generalized error distribution; $\beta_{u}>0(u=0,1,\cdots m)$, $\alpha_{v}>0(v=1,\cdots n)$, $\sum_{u=1}^{m}\beta_{u}+\sum_{v=1}^{n}\alpha_{v}<1$. The GARCH model uses the m-order moving average (ARCH) term of the squared residual sequence $\left\{a_{t}^{2}\right\}$ and the n-order autoregressive (GARCH) term of the sequence $\left\{h_{t}\right\}$ to fit the current heteroscedasticity value, and n and m reflect the order of the GARCH term as well as the ARCH term, respectively. Obviously, the ARCH(m) model is a special case of the GARCH(m,n) model. In particular, when $h_{t}$ is constant and $\left\{e_{t}\right\}$ obeys the Gaussian distribution, the GARCH model is equivalent to the ARMA model.

## 4. Results and Discussions

In this section, the establishment process of the proposed model is described in detail, and the performance of the proposed Kalman-ARIMA-GARCH model is demonstrated by comparison with the Kalman-ARIMA model. All the simulations are carried out in MATLAB 2011a.

### 4.1. Data Preprocessing

**Figure 6:**
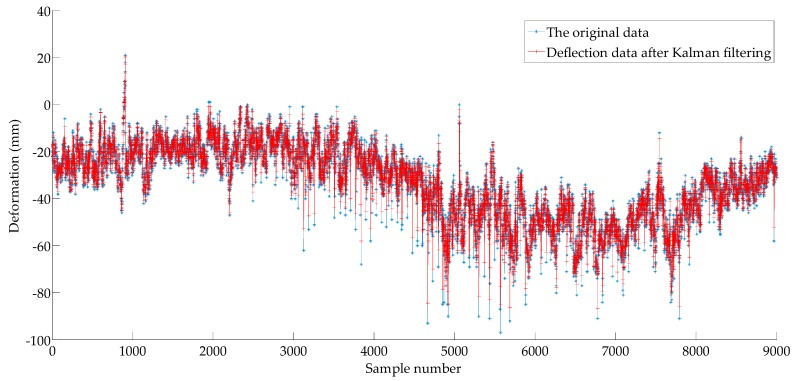
The deflection sample series after the Kalman filter.

A collection of deformation data of mid-span of Caijia Jialing River Bridge measured by GNSS (including 9000 samples) is adopted to examine the effectiveness and reliability of the proposed method. The sampling period is 10 s. Due to the influence of random errors from light, temperature, and the sensors, the variation characteristics of little deformation with significant fluctuation often appear in the deformation monitoring. In this paper, the Kalman filter is used to de-noise the sample data. According to [39], there is a positive correlation between the measurement noise variance and the estimation performance of the filter, and a negative correlation between the system noise variance and the filter performance, in this study, R=0.3, Q=1.0. The time series processing by the Kalman filter is called as $\left\{X_{1t}\right\}$, which is illustrated in Figure 6.

As shown in Figure 6, the Kalman filter can effectively eliminate random noise, and the fidelity of the data after filtering is satisfactory.

**Figure 7:**
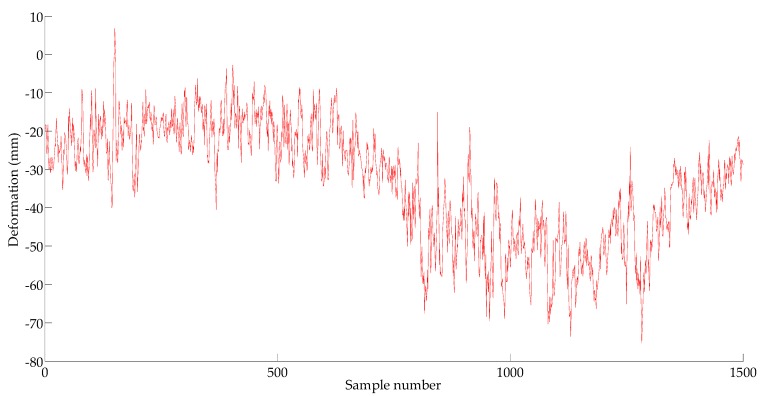
Deformation time series {*X*_2*t*_}.

According to [7,8,10], a single prediction step is set to one minute. If the prediction is directly carried out by using the time series $\left\{X_{1t}\right\}$, the model needs six steps ahead for each forward prediction of 1 min. It is obvious that too many prediction steps will affect the prediction accuracy of time series models. In this study, $\left\{X_{1t}\right\}$ is processed through the average value method every minute. In this way, the advanced 1 min prediction model needs only one step. Compared with the approach of the simple adjustment of the data sampling frequency, the maximum deformation value in the 60 s is retained while the GPS performance is guaranteed. The sequence $\left\{X_{2t}\right\}$ (including 1500 samples) after averaging is shown in Figure 7. Here, the time series is divided into two parts: the 1st–1000th sampling points for training and the subsequent 1001th–15000th data for testing. 

### 4.2. Model Establishment

In this section, the detailed modeling process of ARIMA-GARCH is presented.

#### 4.2.1. Test for Stationary of Time Series

**Figure 8:**
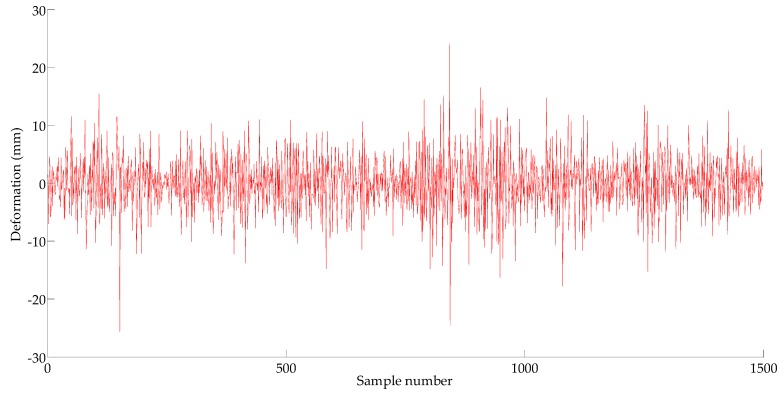
Time series {*X*_3*t*_}.

The runs test is adopted to test the stationarity of time series $\left\{X_{2t}\right\}$. As the result shows there is nonstationarity in $\left\{X_{2t}\right\}$, ARIMA(p,d,q) is used to fit the sequence $\left\{X_{2t}\right\}$. The 1st–1000th sampling points are subjected to first order differencing. Data processing by first difference is recorded as $\left\{X_{3t}\right\}$ in Figure 8. The runs test result of $\left\{X_{3t}\right\}$ shows stationarity, thus, in the ARIMA (p,d,q) model, the order of the difference is equal to one.

#### 4.2.2. Model Identification

**Figure 9:**
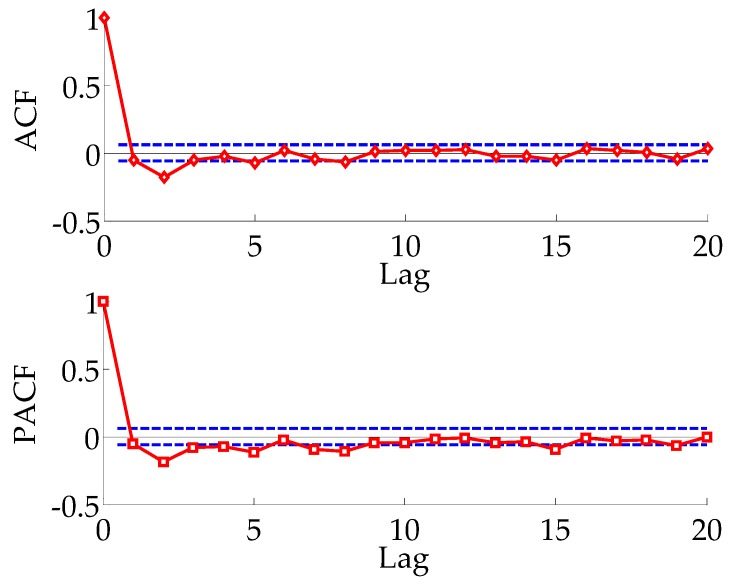
The autocorrelation (ACF) and partial ACF (PACF) for the {*X*_3*t*_} series.

Figure 9 shows analysis results of $\left\{X_{3t}\right\}$ by autocorrelation (ACF) and partial ACF (PACF). As seen from Figure 9, both ACF and PACF exhibit tails of infinite shock. The ARMA model is established to fit $\left\{X_{3t}\right\}$, which is equivalent to establishing an ARIMA(p,1,q) model fitting sequence $\left\{X_{2t}\right\}$.

#### 4.2.3. Model Order Determination

The Akaike information criterion (AIC) is used to determine the value of the autoregressive order p and moving average order q in ARIMA(p,1,q). The mathematical form of the AIC criterion is given by

(16) $$\mathrm{AIC}=-2\ln(c_{1})+2(c_{2}),$$

where $c_{1}$ is the maximum likelihood, and $c_{2}$ is the number of independent parameters.

By comparison, the model fitting is most reasonable when p=3, q=3, thus, the most suitable non-stationary ARIMA(p,d,q) model is the ARIMA(3,1,3) model.

#### 4.2.4. Parameter Estimation

Maximum likelihood estimation is used to estimate the parameters of the ARIMA(3,1,3) model, the equation of ARIMA(3,1,3) model is obtained by

(17) $$\begin{array}{ll} X(t) & =-0.3620X(t-1)+0.4762X(t-2)+0.3935X(t-3)-0.5077X(t-4)+a(t)+0.2128a(t-1)\\ & -0.4317a(t-2)-0.6995a(t-3) \end{array}$$

#### 4.2.5. Test of Heteroscedasticity

**Figure 10:**
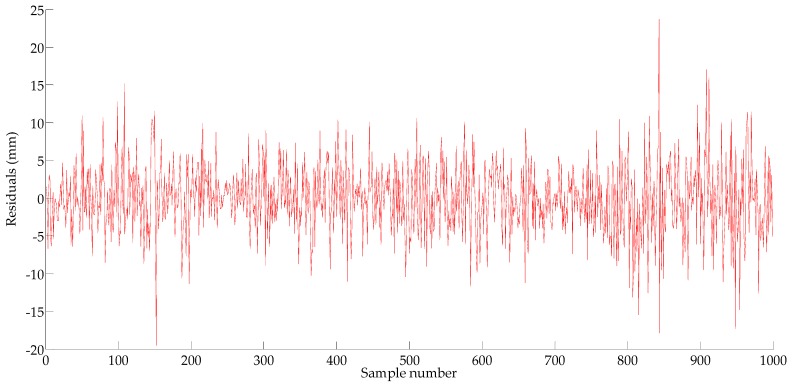
The residual error of autoregressive integrated moving average (ARIMA).

In this study, the residual plot test and the Lagrange multiplier verify (ARCH-LM) are used to examine the heteroscedasticity. As shown in Figure 10, the aggregation of the residuals indicates that the residuals of the ARIMA(3,1,3) model may have heteroscedasticity. If there is heteroscedasticity, the GARCH model would be established, if not, the prediction would be made by the mean Equation (17).

#### 4.2.6. Establishment of the GARCH Model

The results show that the residuals of the ARIMA(3,1,3) model exhibit heteroscedasticity, in which case, the GARCH model should be established. As the GARCH(1,1) model describes heteroscedasticity concisely and has a satisfactory fitting performance, it is considered as the benchmark model [40]. Therefore, this study established the GARCH(1,1) model, and it is found that the GARCH(1,1) model based on *t* distribution has the best fitting effect through the comparison of the optimal principle. Use the maximum likelihood estimation to determine the parameters of the ARCH term and the GARCH term, the GARCH model is obtained by

(18) $$h_{t}=3.325\times 10^{-4}+0.7761a_{t-1}^{2}+0.2109h_{t-1},$$

where the coefficient of the ARCH term and the GARCH term are greater than zero and satisfy the condition $\sum_{u=1}^{m}\beta_{u}+\sum_{v=1}^{n}\alpha_{v}<1$. In this way, a prediction model of ARIMA(3,1,3)-GARCH(1,1) is established, where Equation (17) is the mean equation, and Equation (18) is the variance equation.

### 4.3. Recursive Time Series Model

When utilizing a recursive time series model for advanced multi-step prediction, the parameters of the model are updated by the new predictive values after obtaining the prediction of the next moment by iteration. Then a new model including prediction information is obtained, and then used to predict the next step by the new model. The detailed procedure is described as follows:

Step 1: Modeling $\left\{X_{2t}(1),X_{2t}(2),\cdots ,X_{2t}(1000)\right\}$ by following Section 4.2.1, Section 4.2.2, Section 4.2.3, Section 4.2.4, Section 4.2.5 and Section 4.2.6, and obtain the model equation. Realize one-step prediction and get the predictive value $\bar{X_{2t}}(1)$.

Step 2: Re-estimate the parameters of the model equation by $\left\{X_{2t}(2),\cdots ,X_{2t}(1000),\bar{X_{2t}}(1)\right\}$ (follow Section 4.2.4, Section 4.2.5 and Section 4.2.6), then obtain the new model equation, including the predictive information and make a further prediction, after that, complete five-step prediction in the same way.

Step 3: Make a five-step prediction for $\left\{X_{2t}(2),X_{2t}(3),\cdots ,X_{2t}(1001)\right\}$ by repeating the above two modeling steps until the five-step of $\left\{X_{2t}(496),X_{2t}(497),\cdots ,X_{2t}(1495)\right\}$ is achieved in the same way.

### 4.4. Evaluation Criteria

To quantitatively evaluate the accuracy and stability of proposed model, the mean absolute error (MAE), mean relative percentage error (MRPE), root mean square error (RMSE) and root mean square relative error (RMSRE) are utilized in this study, the calculation formulas are shown as follows:

(19) $$MAE=\frac{1}{n_{te}}\sum_{i=1}^{n_{te}}\left|x_{i}-\bar{x_{i}}\right|,$$

(20) $$MRPE=\frac{1}{n_{te}}\sum_{i=1}^{n_{te}}\left|\frac{x_{i}-\bar{x_{i}}}{x_{i}}\right|\times 100\%,$$

(21) $$RMSE=\sqrt{\frac{1}{n_{te}}\sum_{i=1}^{n_{te}}{\left(x_{i}-\bar{x_{i}}\right)}^{2}},$$

(22) $$RMSRE=\sqrt{\frac{1}{n_{te}}\sum_{i=1}^{n_{te}}{\left(\frac{x_{i}-\bar{x_{i}}}{x_{i}}\right)}^{2}},$$

where $x_{i}$ and $\bar{x_{i}}$ represent the measured data and the predicted data at the time *t*, respectively; $n_{te}$ is the number of the data for performance evaluation.

### 4.5. Forecasting Results

**Figure 11:**
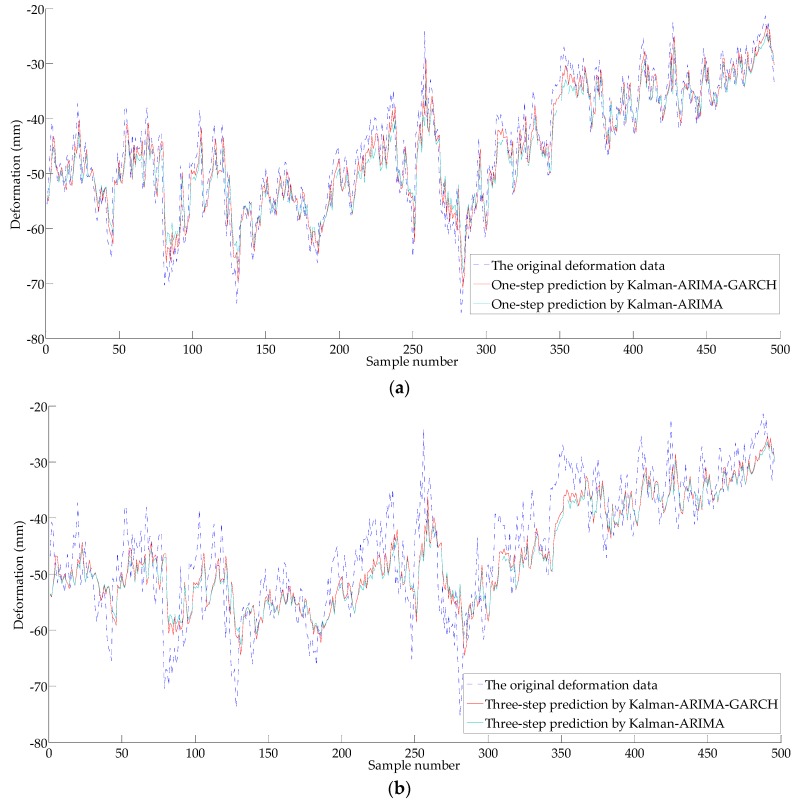
Results of the predictions for the original deformation series {*X*_2*t*_} by the ARIMA and the autoregressive conditional heteroscedasticity model (GARCH): (**a**) One-step prediction; (**b**) Three-step prediction.

**Figure 12:**
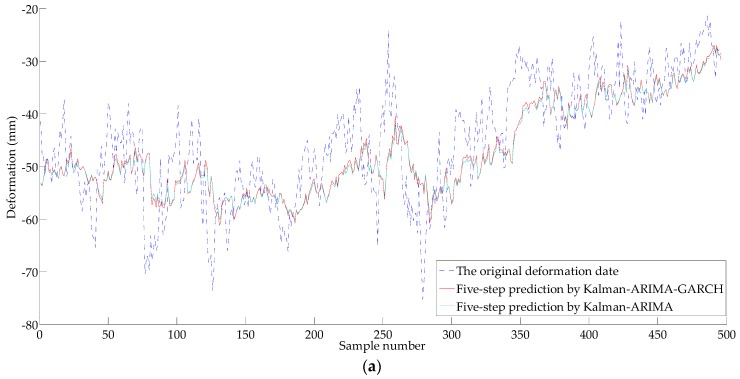

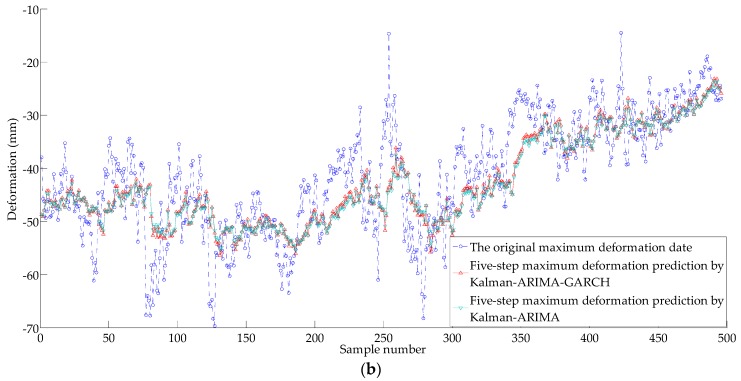
Results of the predictions for the original deformation series {*X*_2*t*_} by the ARIMA and the GARCH: (**a**) Five-step prediction; (**b**) Five-step maximum deformation prediction.

For time series $\left\{X_{2t}\right\}$, the ARIMA model (without considering of heteroscedasticity) and the ARIMA-GARCH model (consider heteroscedasticity) are used to predict the deformation, and the prediction results are shown in Figure 11 and Figure 12. The error analysis results are shown in Table 1, Table 2 and Table 3.

As shown in Table 1, the prediction accuracy of the Kalman-ARIMA-GARCH model is satisfactory with only 3.402 mm of MAE and 10.59% of RMSER. With the increase of prediction step, the degradation of the accuracy appears, as MAE of three-step prediction is 5.098 mm, and RMSRE is 15.78%. The MAE of five lead forecast is 5.847 mm, and the RMSRE is 18.19%. Compared with the Kalman-ARIMA model, the Kalman-ARIMA-GARCH model has obviously better forecasting performance.

As can be seen from Figure 11 and Figure 12, the prediction results have lag characteristics. The property has little influence on the prediction results by sustained growth (or decrease) in deformation, however, the prediction results will bring obvious error when the deformation mutation occurs. The main reason for the hysteresis is that the prediction is realized by the historical deformation sequence, so it is closely related to the historical information. Also, nonlinear behavior of a variable is simplified to a linear model by the time series method, which causes a certain gap between simplify and the actual situation; a recursive multi-step prediction model based on the historical deformation sequence produce accumulative errors. As shown in Figure 11 and Figure 12, as the GARCH model is a nonlinear model, it can explain part of the nonlinear characteristics (heteroscedasticity). As shown by Table 1, Table 2 and Table 3, the prediction effect of ARIMA-GARCH is better than that of the linear ARIMA model.

Based on the success of the five-step prediction by using GARCH model, the maximum deformation value of five minute ahead can be predicted. The calculation method is as follows: (1) according to the recursive time series modeling method, obtain five minute ahead average prediction value; (2) calculate the correlation coefficient *H* of $\left\{X_{1t}\right\}$ between mean value of 60 s with the maximum value according to the least square method, in this study, *H* = 0.97; and (3) revise the predicted values obtained by step (1) according to *H*, and obtain the maximum predictive value in the five minutes. The comparison between the predicted maximum value and the measured maximum one are shown in Figure 12b and Table 3. The MAE of the proposed method is 5.666 mm, and the RMSRE is 21.07%. Compared with Kalman-ARIMA model, the prediction result of Kalman-ARIMA-GARCH model is more reliable.

The prediction model adopted in this paper is composed of a linear ARIMA model (explaining subject part) and nonlinear GARCH model (explaining residuals part), which can only explain partial nonlinear characteristics. To further explain this nonlinearity, the nonlinear model can be introduced to explain the main part, while the residual term explained by the GARCH model. The specific form can be expressed as

(23) $$X\left(t\right)=f\left(X(t-1),\cdots X(t-p)\right)+a\left(t\right)+\theta \left(B\right)\times a_{t},$$

where X(t), a(t), p and θ(B) are consistent as defined in Section 3.3; a(t) can be explained by the GARCH model, f(g) can be interpreted by the kernel density estimation of the Gaussian kernel function using a window function.

## 5. Conclusions

In this paper, the residuals of the ARIMA model of the deformation sequence is verified, and it is found that the deformation time series has an obvious heteroscedasticity effect. To address this difficulty, the GARCH model is introduced to predict the deformation and compared with the traditional linear ARIMA model. Some concluding remarks can be summarized as follows.

(1) Table 1, Table 2 and Table 3 indicate that for non-stationary data with embedded heteroscedasticity, the prediction accuracy of GARCH model is superior to the ARIMA model, the prediction error analysis results of the two models show that the GARCH model has certain advantages under the background that the error appeared in the heteroscedasticity condition. The GARCH model captures this nonlinear information to a certain extent.

(2) The overall actual predictive performance of the GARCH model is not inferior of ARIMA model in the mean sense, and the GARCH model has some edges over ARIMA in a few indicators. In particular, the GARCH model makes up for the imperfections that the ARIMA model is not satisfied with the assumption of constant variance. Thus, GARCH has advantages from the standpoint of theoretical rigor.

(3) The GARCH model provides a new feasible method for bridge deformation prediction. The theories are well designed and established, and the practical prediction result is also satisfactory. The results can lay a foundation for the early warning of bridge health monitoring system based on sensor technology.

The GARCH model alone is not fully applicable and reasonable to explain the whole nonlinear characteristics of deformation time series. More relevant models should be analyzed for the further study.

## Figures and Tables

**Table 1:** Analysis of the predictions given in Figure 11a.

Indices	Kalman-ARIMA	Kalman-ARIMA-GARCH	Comparison Results	Improvements
MAE of average deformation prediction (mm)	4.407	3.402	1.005	22.80%
MRPE of average deformation prediction (%)	10.16	7.96	2.20	21.65%
RMSE of average deformation prediction (mm)	5.317	4.341	0.976	18.36%
RMSRE of average deformation prediction (%)	13.20%	10.59%	0.0261	19.77%

MAE: mean absolute error; MRPE: mean relative percentage error; RMSE: root mean square error; RMSRE: root mean square relative error; ARIMA: autoregressive integrated moving average model; GARCH: generalized autoregressive conditional heteroskedasticity.

**Table 2:** Analysis of the predictions given in Figure 11b.

Indices	Kalman-ARIMA	Kalman-ARIMA-GARCH	Comparison Results	Improvements
MAE of average deformation prediction (mm)	5.887	5.098	0.789	13.40%
MRPE of average deformation prediction (%)	13.99	12.02	1.97	14.08%
RMSE of average deformation prediction (mm)	7.890	6.448	1.442	18.28%
RMSRE of average deformation prediction (%)	17.69	15.78	1.91	10.80%

**Table 3:** Analysis of the predictions given in Figure 12.

Indices	Kalman-ARIMA	Kalman-ARIMA-GARCH	Comparison Results	Improvements
Five step average deformation prediction				
MAE of average deformation prediction (mm)	6.505	5.847	0.658	10.12%
MRPE of average deformation prediction (%)	14.95	13.91	1.04	6.96%
RMSE of average deformation prediction (mm)	7.885	7.293	0.592	7.51%
RMSRE of average deformation prediction (%)	20.11	18.19	1.92	9.55%
Five step maximum deformation prediction				
MAE of maximum deformation prediction (mm)	6.151	5.666	0.485	7.88%
MRPE maximum deformation prediction (%)	16.13	14.88	1.25	7.75%
RMSE of maximum deformation prediction (mm)	7.811	7.156	0.655	8.39%
RMSRE of maximum deformation prediction (%)	23.07	21.07	2.00	8.67%
